# High-Performance Organic Photodetectors by Introducing a Non-Fullerene Acceptor to Broaden Long Wavelength Detective Spectrum

**DOI:** 10.1186/s11671-019-3033-8

**Published:** 2019-06-11

**Authors:** Genjie Yang, Zijun Wang, Yuxiang Duan, Dan Zhao, Junsheng Yu

**Affiliations:** State Key Laboratory of Electronic Thin Films and Integrated Devices, School of Optoelectronic Science and Engineering, University of Electronic Science and Technology of China (UESTC), Chengdu, 610054 China

**Keywords:** Organic photodetectors, Non-fullerene acceptor, Surface morphology, UV-Vis absorption, Full visible light photodetection

## Abstract

We demonstrate the broadband visible organic photodetectors (OPDs) by introducing a non-fullerene acceptor of 3,9-bis(2-methylene-(3-(1,1dicyanomethylene)-indanone))-5,5,11,11-tetrakis(4-hexylphenyl)-dithieno[2,3d:2,3′-d′]-s-indaceno[1,2-b:5,6-b′]dithiophene (ITIC) into the bulk heterojunction (BHJ) based on a conventional system of poly(3-hexylthiophene-2,5-diyl) (P3HT):[6,6]-phenyl C71-butyric acid methyl ester (PC_71_BM) .The resultant OPDs exhibit a specific detectivity beyond 10^12^ Jones in the whole visible region ranged from 380 nm to 760 nm, and the highest detectivity reaches 2.67 × 10^12^ Jones at 710 nm. UV-Vis absorption spectrum, steady-state photoluminescence, atomic force microscopy, and space-charge-limited current property were applied to analyze the film characteristics of obtained OPDs. Owing to the long-wavelength absorption band of ITIC, the spectral photodetection range has been broadened effectively, and better film morphology, more effective energy transfer, and the reduced electron mobility in the active layer are responsible for the excellent photodetection capability. The proposed scheme provides a reliable strategy for implementing high-performance broadband visible OPDs.

## Introduction

Visible light, as part of electromagnetic spectrum that can be directly perceived by human vision (380–780 nm), plays an important role in daily life and industrial production [1]. Visible light remote sensing is the most commonly used in aerial photographic reconnaissance. Color image sensing is also mostly based on visible light, etc. [2]. As a bridge between the optical signal and electrical signal, photodetector plays an irreplaceable role in the above applications, thus causing extensive and continuous attention [3]. Therefore, the research of high-performance visible photodetector is imperative and of great significance. Compared with traditional inorganic photodetectors, organic photodetectors (OPDs) have attracted tremendous attention for applications in flexible and portable electronic applications due to their flexibility, tunable absorption, lightweight, large-area detection, and low cost of preparation [4]. In recent years, although OPDs have made some achievements in such aspects as high-external quantum efficiency [5], low dark current density [6] and high detectivity [7], there are only a few research attempts to investigate high-performance broadband OPDs with full visible photodetection until now.

The efficient light harvesting and broad absorption range are of crucial importance in broadband OPDs. Therefore, many donor and acceptor materials with different band gaps have been developed and many classical donor/acceptor heterojunction systems have been constructed in the course of past research [8]. Among them, poly(3-hexylthiophene) (P3HT):phenyl-C71-butyric acid methyl ester (PC_71_BM) bulk heterojunction (BHJ) has been widely studied in organic photovoltaic devices, on account of its relatively high-carriers mobility, stable performance, simple structure, low cost, and mature preparation process [9, 10]. Nevertheless, although the spectral response of P3HT:PC_71_BM covers 400–600 nm, it is not wide enough to constitute full visible photodetection, because of the absence of the long-wave region. Therefore, it is necessary to find an effective method to expand the spectral response range of P3HT:PC_71_BM conventional system. Similar to organic solar cells (OSCs) [11, 12], introducing a third material into the active layer is one of the most efficient and simple methods to fulfill the broadband OPDs with extended photodetection range and excellent performance [13]. For example, Rauch et al. developed the P3HT:PC_71_BM BHJ where PbS quantum dots as the introducing component, which successfully extended the detective range of OPDs to 1800 nm [14]. Mario Caironi et al. developed the T1:P3HT:PC_71_BM OPDs with broadband response of 360–680 nm by introducing a middle-wavelength-absorption electron donor T1 [15].

Recently, a new class of non-fullerene electron acceptors has shown high absorption coefficients and excellent electrical properties, yielding widespread concern in the research of photovoltaic devices [16, 17]. Compared with conventional fullerene derivatives acceptors, non-fullerene acceptors have diversified and strong absorption, so they are the better options to introduce into the traditional system as the third component [18]. For example, Tan et al. developed a ternary acceptor blending device by doping 3,9-bis(2-methylene-(3-(1,1dicyanomethylene)-indanone))-5,5,11,11-tetrakis(4-hexylphenyl)-dithieno[2,3d:2,3′-d′]-s-indaceno[1,2-b:5,6-b′]dithiophene (ITIC) in the PBDTBDD:PC_60_BM blend to achieve perfect complementary absorption and high PCE of 10.36% [19]. Furthermore, the distinctive feature of ITIC is the long-wave spectral response of 600–800 nm, compared with the short and medium wave response inherent in traditional fullerene derivatives. Therefore, ITIC may be suitable for combination with P3HT:PC_71_BM BHJ with the response of 400–600 nm, which can extend the photodetection range to the long-wave range to realize the effective photodetection of full visible spectrum continuously.

Hence, in this work, ITIC is firstly introduced into P3HT:PC_71_BM conventional system to form broadband OPDs. Compared with the control P3HT:PC_71_BM OPDs, the ternary blends system achieves a wider spectral response. Meanwhile, by tuning the ratios of ITIC and PC_71_BM respectively, the broadband OPDs covering the full visible band from 380 nm to 760 nm are obtained, compared with the original photodetection band of 380–620 nm. Moreover, due to the wider light harvesting region, better film morphology, more effective energy transfer, and the lower dark current, the optimizing OPDs exhibited a high detectivity of 2.12 × 10^12^ Jones and 2.67 × 10^12^ Jones at 560 nm and 710 nm, respectively.

## Methods

**Fig. 1:**
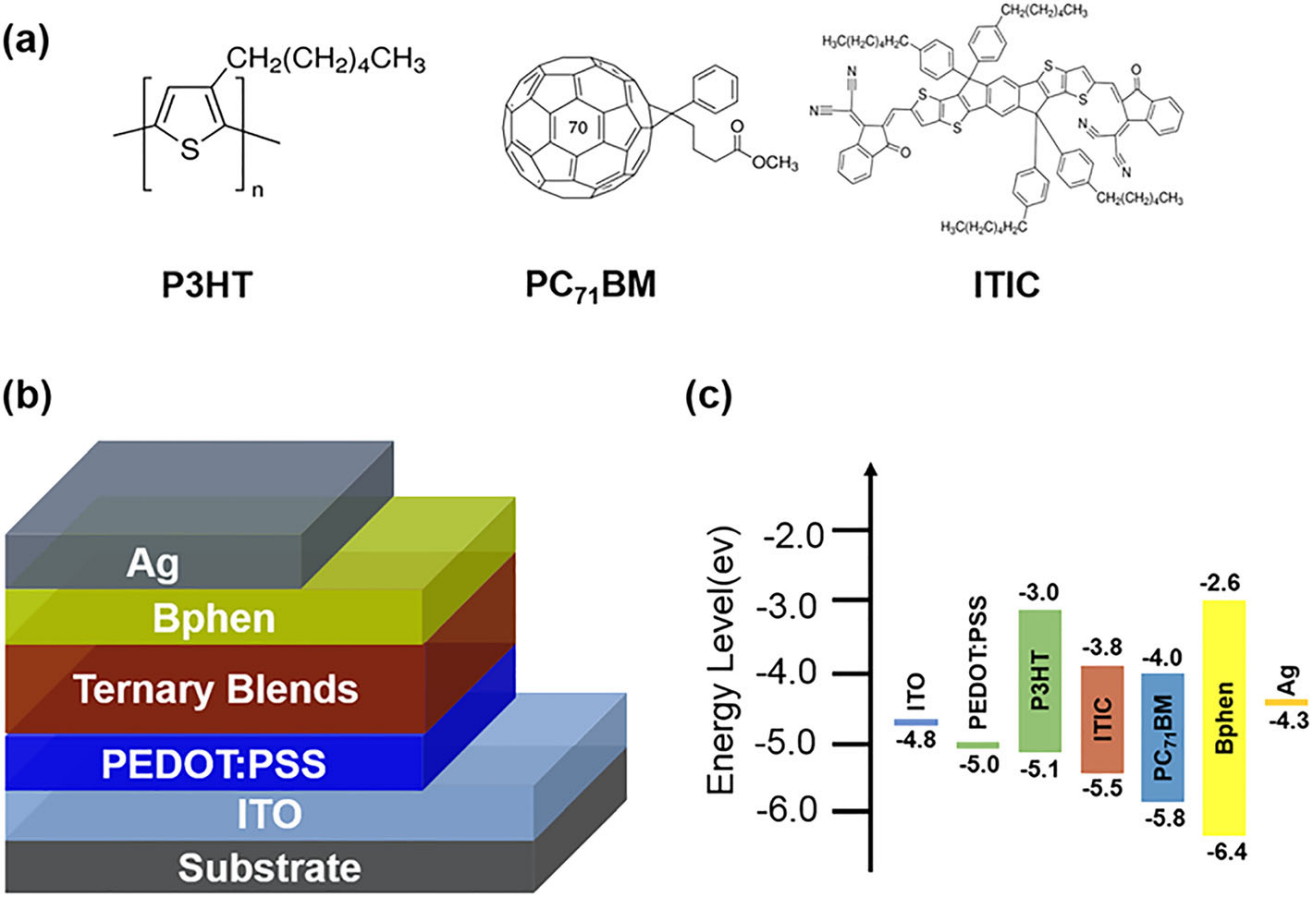
**a** Chemical structures of active layer materials. **b** Device structure of OPDs. **c** Energy level diagram of OPDs

The molecular structures of active layer materials used in this work are shown in Fig. 1a, and the broadband OPDs structure of indium tin oxide (ITO)/poly(3,4-ethylenedioxythiophene):polystyrene sulfonate (PEDOT:PSS) (45 nm)/P3HT:PC_71_BM:ITIC (100 nm)/Bphen (5 nm)/Ag (80 nm) is depicted in Fig. 1b. The energy levels of active layer materials in broadband OPDs are shown in Fig. 1c. The lowest unoccupied molecular orbital (LUMO) and the highest occupied molecular orbital (HOMO) levels of P3HT, ITIC, and PC_71_BM follow a normative cascade alignment, which indicates the potential efficient charge transport pathway among them. Bphen is used as a buffer layer to improve charge carrier transport ability and reduce the quenching of photo excitons at the interface between the active layer and the cathode [20]. Otherwise, the HOMO of Bphen is higher than active materials, which can be used as a hole-blocking layer to reduce dark current under reverse bias.

Before starting the OPDs fabrication, ITO substrates were consecutively cleaned in ultrasonic bath for each 10 min with water-detergent solution, acetone solvent, deionized water, and IPA solvent, respectively [21]. After dried in the oven, these ITO substrates were treated with oxygen plasma for 20 min. Then, PEDOT:PSS was spin-coated at 3000 rpm for 60 s on ITO substrates. After thermal annealing at 150 °C for 20 min, the substrates were moved into a high-purity glovebox (O_2_, H_2_O < 1 ppm). P3HT, PC_71_BM, and ITIC were dissolved in chlorobenzene with different mass ratios. The total concentration of these materials was fixed at 30 mg ml^−1^, and the blend mass ratio of donor (P3HT) and acceptors (PC_71_BM, ITIC) was fixed at 1:1. Active layer solutions were spin cast on the top of PEDOT:PSS layer at 2000 rpm for 60 s. Subsequently, the blend films were annealed at 120 °C for 10 min. Followed by the deposition of Ag as anode at a deposition speed of 5 Å S^−1^. The active area of these OPDs was 0.02 cm^2^.

### Device Characterization

The ultraviolet-visible (UV-Vis) absorption was measured by using a Shimazu UV1700 UV-Vis spectroscopy system. The steady-state photoluminescence (PL) was measured by using a Hitachi F-7000 PL spectroscopy. Surface morphologies of active layers were characterized by atomic force microscope (AFM, AFM 5500, Agilent, Tapping Mode, Chengdu, China). A light source was used as an AM 1.5 G solar simulator with an illumination power of 100 mW cm^−2^. The current density-voltage (*J-V*) curves of OPDs in the dark and under illumination were measured with a Keithley 4200 programmable voltage-current source. The EQE spectra were obtained under a xenon lamp light passing through a monochromator. All parameters were measured at room temperature (*T* = 300 k).

## Results and Discussion

### Characterization of Active Layers

**Fig. 2:**
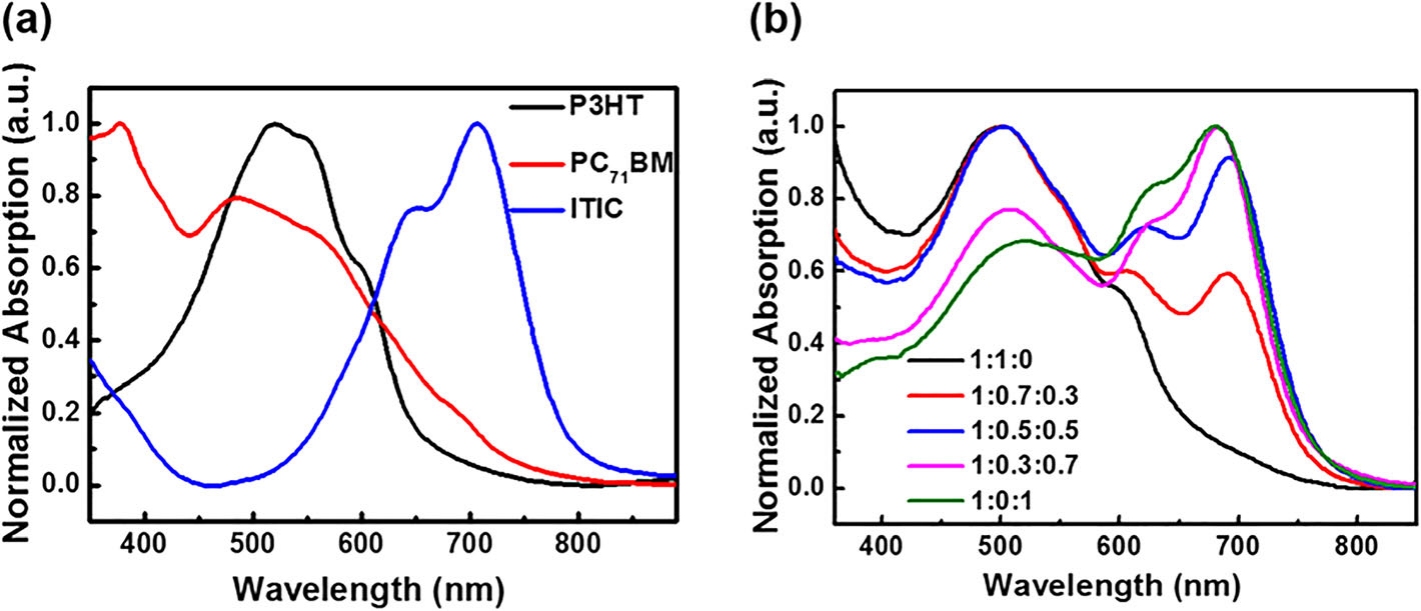
**a** Absorption of pure P3HT, PC_71_BM, and ITIC films. **b** Absorption spectra of active layers with different ratios

The absorption spectra of pure P3HT, PC_71_BM, and ITIC films are displayed in Fig. 2a. PC_71_BM can absorb the short wavelength from 350 nm to 550 nm. P3HT can utilize light in the middle wavelength from 450 nm to 600 nm. And the non-fullerene electron acceptor, ITIC, can realize the absorption from 600 nm to 800 nm. Obviously, these three active layer materials achieve a favorable complementary in full visible spectrum. So, the blend films have the superexcellent potential of realizing full visible photodetection. Moreover, the absorption spectra of the active layers (P3HT:PC_71_BM:ITIC) with different ratios are depicted in Fig. 2b. P3HT:PC_71_BM films show favorable light absorption capacity from 400 nm to 600 nm, but there is almost no absorption in the long-wave region after 600 nm. After introducing ITIC, a new absorption peak is generated from 600 nm to 750 nm because of the contribution of ITIC. With the gradual increase of the incorporation of ITIC, the absorption capacity of the blend films in the long wavelength gradually increases, which is beneficial to broaden the long wavelength detective spectrum of P3HT:PC_71_BM control system. Furthermore, the absorption intensity at short and long wavelengths can be effectively tuned by varying the ratios of PC_71_BM and ITIC. In particular, balanced absorption intensity is achieved when the mass ratio of the active layer is 1:0.5:0.5, which is obviously beneficial to equilibrate photodetection of OPDs in short and long wavelengths simultaneously and realize the broadband OPDs with full visible photodetection.

**Fig. 3:**
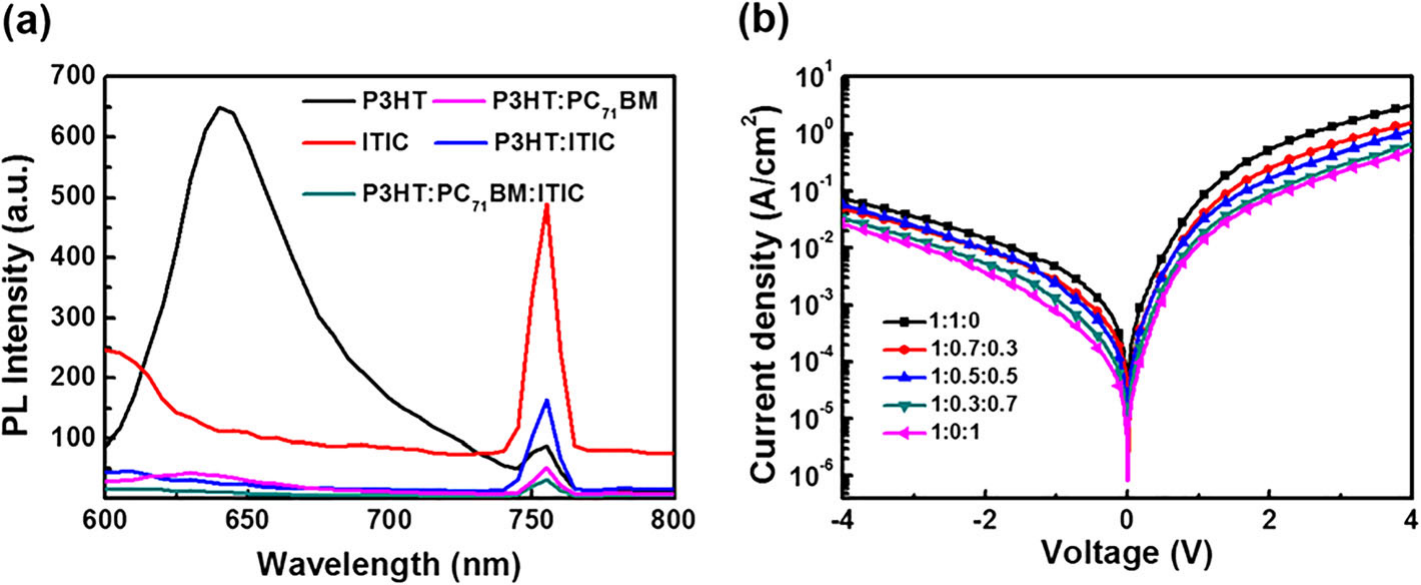
**a** PL spectra of films under 500 nm light excitation. **b** *J-V* characteristics of electron-only devices

To investigate the influence of introducing ITIC on the energy transfer in active layers, steady-state photoluminescence (PL) tests were performed. As shown in Fig. 3a, when excited by 500 nm light, the neat P3HT and ITIC films exhibit PL peaks at 640 nm and 760 nm, respectively. Compared with neat P3HT film, the PL intensity of P3HT is greatly quenched in P3HT:ITIC film, which indicates the existence of an energy transfer between P3HT and ITIC [22]. Similarly, the PL emission of P3HT is greatly quenched by doping with PC_71_BM in the P3HT:PC_71_BM film, which indicates analogical efficient energy transfer between P3HT and PC_71_BM. Moreover, when introducing ITIC to the P3HT:PC_71_BM blend film, the PL intensity is almost completely quenched, and the PL curve of ternary blend film is below all other curves. It means that both ITIC and PC_71_BM can coordinately transfer the energy in ternary films. It is concluded that the energy transfer efficiency of ternary films is better than that of binary films. Combined with the fact that the former has a wider light absorption range than the latter to capture more photons to contribute to the photocurrent, it indicates P3HT:PC_71_BM:ITIC OPDs may have higher photocurrent than P3HT:PC_71_BM OPDs in theory.

To investigate the influence of charge carrier transport properties by the introduction of ITIC, the space-charge-limited current (SCLC) model was adopted for mobility quantify. The electron-only devices were fabricated with the structure of ITO/ZnO (30 nm)/P3HT:PC_71_BM:ITIC (100 nm)/Bphen (5 nm)/Ag (80 nm). The SCLC is described by the Mott–Gurney equation [23]:

1 $$ J=\frac{9}{8}{\varepsilon \varepsilon}_0\mu \frac{V^2}{d^3} $$

where *ε*_*0*_ is the vacuum permittivity, *ε* is the relative permittivity of the organic materials, *μ* is the charge carrier mobility, *V* is the applied voltage, and *d* is the thickness of the active layers. *J-V* characteristics in dark condition for the electron-only devices with different active layers are shown in Fig. 3b. According to Eq. (1), the electron mobility of devices with different ratios are 1.48 × 10^−3^ cm^2^ V^−1^ s^−1^, 8.92 × 10^−4^ cm^2^ V^−1^ s^−1^, 7.89 × 10^−4^ cm^2^ V^−1^ s^−1^, 4.75 × 10^−4^ cm^2^ V^−1^ s^−1^, and 4.43 × 10^−4^ cm^2^ V^−1^ s^−1^, respectively. With the increase of the proportion of ITIC, the electron mobility of device decreases significantly since the electron mobility of ITIC is lower than PC_71_BM [24], which may cause the dark current of the OPDs to decrease after the introducing of ITIC [25].

**Fig. 4:**
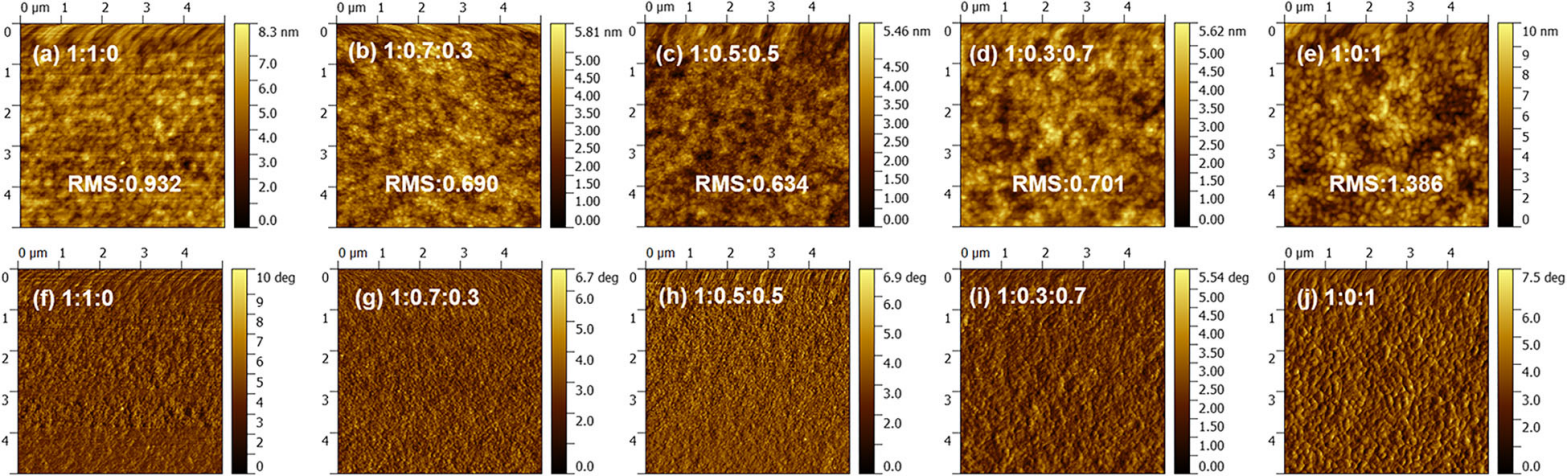
AFM height images (**a**–**e**) and phase images (**f**–**j**) of P3HT:PC_71_BM:ITIC active layers with various ratios

For OPDs, the surface morphology of the active layer has a great influence on charge transport and exciton dissociation. An active layer with favorable surface morphology can inhibit the charge recombination and improve photocurrent [26]. Hence, the surface morphologies of active layers with different ratios are investigated by atomic force microscopy (AFM), which are depicted in Fig. 4. According to the height image, the surface of the P3HT: PC_71_BM: ITIC (1:1:0) film is a little rough and the root-mean-square (RMS) roughness is about 0.932 nm. From the phase image, we can see that the arrangement of molecules is not completely uniform and orderly. After doping some portion ITIC to the blend (1:0.7:0.3, 1:0.5:0.5, 1:0.3:0.7), the surface morphology of the active layer changes greatly and the RMS roughness goes down to 0.690 nm, 0.634 nm, and 0.701 nm, respectively. The variation of RMS may be attributed to the changed aggregation state, as can be seen from phase diagrams. Compared with the P3HT:PC_71_BM binary film, the ITIC doped blend films exhibits the smoother surface and more ordered molecular arrangement. However, when the ratio of the blend becomes 1:0:1, the RMS roughness increases to 1.386 nm and the film morphology is not smooth enough caused by granular undesirable molecular aggregation, which may lead to the increase of charge recombination and low photocurrent. According to the AFM characterization results, the ternary blend films have better morphological characteristics than binary films, which are due to the ordered arrangement of molecules of the two acceptors, reducing the molecular aggregation in the ternary films.

According to the absorption spectra of active layers, the long-wavelength absorption band of introduced ITIC should be able to broaden the long wavelength photodetection range of OPDs effectively. Furthermore, the introduction of ITIC also changes the electrical properties and surface morphology of active layers. From the perspective of SCLC, the introduction of ITIC reduces the electron mobility of the active layer, which obviously would reduce the carrier transport capacity of the devices. This would have the same adverse effect on dark current and photocurrent. However, the introduction of ITIC also allows the active layer to capture more photons from long wavelength to contribute photocurrent, which overcomes the adverse effect of low electron mobility on photocurrent under light condition. Better film morphology and more effective energy transfer in the ternary active layer are also beneficial to the excellent photocurrent. In conclusion, dark current will decrease with the addition of ITIC, while photocurrent will change regularly under the influence of various factors. Therefore, it is necessary to prepare OPDs constructed by active layers with different ratios to determine the high photocurrent and low dark current, so as to achieve excellent photodetection performance.

### Performance of OPDs

**Fig. 5:**
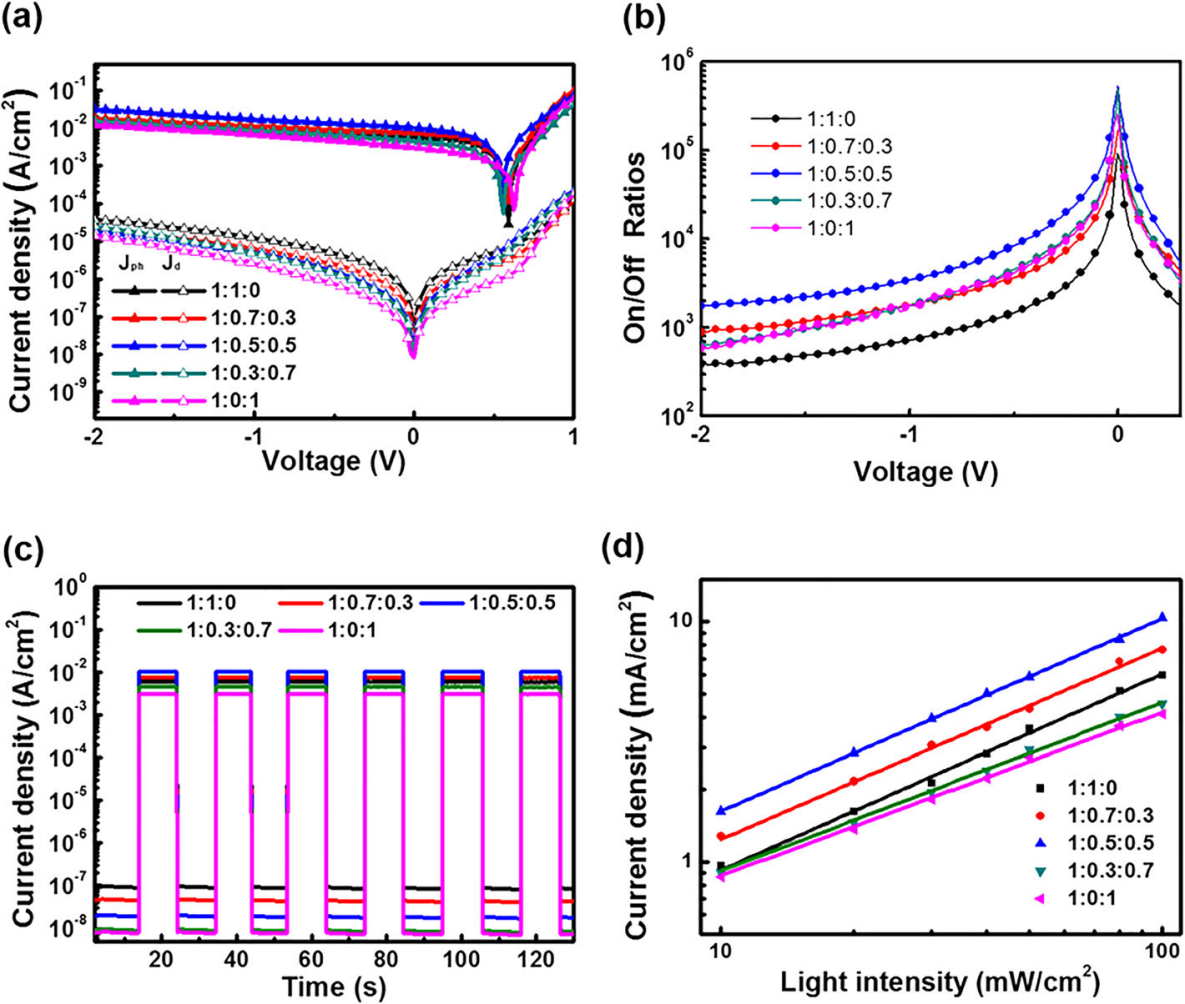
**a** *J*-*V* characteristics of OPDs with various ratios under dark and light conditions. b On/off ratios of OPDs. **c** Response/recovery characteristics of the OPDs under light on/off modulation. **d** *J*_SC_ of OPDs as a function of light intensity

Figure 5 shows electrical performance parameters of OPDs with different ratio of active layers. The *J-V* curves of OPDs under light and dark conditions are presented in Fig. 5a. As shown, the OPDs with different mass ratios of active layer have significantly different photocurrent and dark current. Concretely, as the P3HT:PC_71_BM:ITIC ratio changes from 1:1:0 to 1:0.5:0.5, the photocurrent keeps increasing, which is caused by expanded light harvesting range, efficient energy transfer, and better film morphology in ternary blends. Conversely, as the P3HT:PC_71_BM:ITIC ratio changes from 1:0.5:0.5 to 1:0:1, the photocurrent keeps going down. However, the dark current keeps decreasing as the ITIC ratio increasing, which attribute to reduced electron mobility and unfavorable charge carrier transport caused by of excessive addition of the ITIC. The changing trend of photocurrent and dark current is consistent with the change of film properties caused by the change of ternary ratios of active layers. The on/off ratios characteristics of OPDs are investigated in Fig. 5b. The 1:0.5:0.5 OPDs show the highest on/off ratios in the reverse bias region than the other OPDs, demonstrating a much better switch property, which is due to the highest photocurrent and lower dark current.

Additionally, to make sure the OPDs have a stable and recoverable response ability, the current density as a function of time is shown in Fig. 5c for the broadband OPDs with various ratios. The cyclical current signals were recorded upon the on/off modulation of the light illumination. Each cycle is 20 s with an exposure time of 10 s and the total duration is 120 s. The results show that the current of each OPD increases significantly under illumination and returns to the original level after the light is turned off. It is obvious that these OPDs have stable and repeatable response/recovery characteristics, which is desirable for practical applications [27].

To further investigate the influence of the ITIC ratio on recombination of OPDs in light condition, *J*_SC_ as a function of light intensity is plotted. In general, a power law dependence between *J*_SC_ and *I* can be expressed as *J*_SC_∝*I*^α^. When α approaches 1, bimolecular recombination is relatively weak [28, 29]. As shown in Fig. 5d, the OPDs with the ratio of 1:1:0, 1:0.7:0.3, and 1:0.5:0.5 have the similar α values, which are 0.817, 0.797, and 0.803, respectively. This means that these three OPDs have a similar level of bimolecular recombination. However, due to the introduction of ITIC, more long-wave photons are absorbed in ternary active layers, so that the photocurrent of the OPDs with moderate doping ITIC is greater than that of the P3HT:PC_71_BM OPDs. As further changing the ternary ratios to 1:0.3:0.7 and 1:0:1, the α values drop to 0.713 and 0.680, respectively. This indicates that the large amount of ITIC doping intensifies the recombination and significantly reduces the photocurrent.

**Fig. 6:**
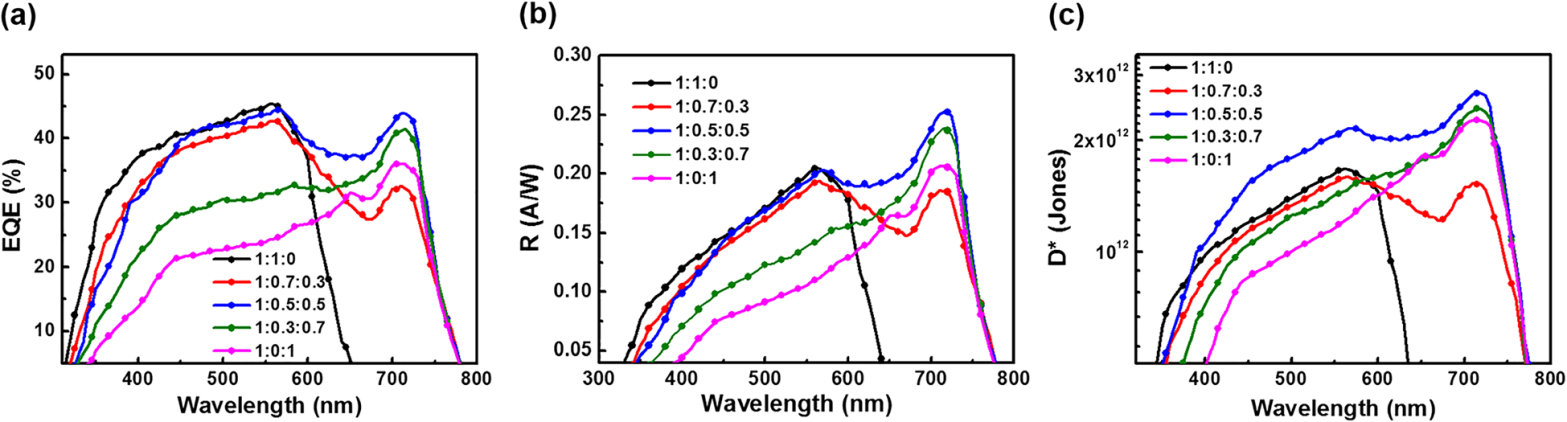
**a** Measured EQE spectra of OPDs with various ratios. **b** Calculated *R* values of OPDs. **c** Calculated D* values of OPDs

In order to describe the spectral response characteristics of the OPDs, the EQE curves of the OPDs with various P3HT: PC_71_BM: ITIC ratios are showed in Fig. 6a. And some parameters of spectral detection performance at different specific wavelengths are listed in Table 1. The device based on binary P3HT:PC_71_BM film shows flat EQE peak covering the ranges of 400–600 nm, attributed to the absorption of P3HT and PC_71_BM. After introducing non-fullerene, ITIC, into P3HT:PC_71_BM, the EQE curve of the broadband OPDs extends to 760 nm, and a new spectral peak from 650 nm to 750 nm is generated. Furthermore, the relative response intensity of the different spectral ranges can be tuned by changing the mass ratios of P3HT, PC_71_BM, and ITIC. From the EQE curves, the synergy between the donor and acceptors at optimal mass ratio, 1:0.5:0.5, balances the EQE of the entire wavelength. The wide and flat EQE curve intuitively shows that the broadband OPDs doped with ITIC effectively extends the continuous optical response range to the long-wave range, covering with whole visible spectrum of 380–760 nm.

**Table 1:** Photodetective performance of obtained OPDs

P3HT:PC_71_BM:ITIC	EQE (%) @		*R* (*A W*^−1^) @		D* (× 10^12^ Jones) @	
	560 nm	710 nm	560 nm	710 nm	560 nm	710 nm
1:1:0	45.34	0.89	0.20	0.01	1.67	0.04
1:0.7:0.3	42.73	32.64	0.19	0.19	1.58	1.53
1:0.5:0.5	43.78	43.90	0.20	0.25	2.12	2.67
1:0.3:0.7	31.46	41.18	0.14	0.23	1.45	2.41
1:0:1	24.41	36.15	0.11	0.21	1.21	2.27

Responsivity (*R*) describes the conversion ability from photons to charge carriers of OPDs, which is used to determine the ability of light response [30]. *R* is calculated as the Eq. (2):

2 $$ R\left(\lambda \right)=\frac{\mathrm{EQE}\left(\lambda \right)q}{hv} $$

where EQE is external quantum efficiency, *q* is the electron charge, *λ* is the wavelength of incident light, *h* is the Planck constant, and *v* is the frequency of light. According to Eq. (2), the trend of *R* is dependent on the EQE and *λ* when the other parameters are constant. The calculated results of *R* values are shown in Fig. 6b and Table 1. Similar to the EQE curves, 1:0.5:0.5 based OPDs obtain higher *R* than other OPDs in both long wavelength and short wavelength range. The *R* values of optimizing broadband OPDs reached 0.21 A W^−1^ and 0.25 A W^−1^ at 560 nm and 710 nm, respectively. The wide *R* curve indicates that the broadband OPDs doped with appropriate amount of ITIC can absorb the incident light of the full visible spectrum evenly and convert it into photocurrent efficiently.

As the most crucial performance parameter of OPDs, the D* is employed to determine the photosensitivity of OPDs. The D* of OPDs can be defined as the Eq. (3):

3 $$ D\ast \left(\lambda \right)=\frac{R\left(\lambda \right)}{{\left(2{qJ}_d\right)}^{1/2}} $$

The calculated results of D* are shown in Fig. 6c. For the control OPDs based on P3HT:PC_71_BM, the detectivity exceeds 1.0 × 10^12^ Jones from 380 nm to 600 nm and reaches 1.67 × 10^12^ Jones at 560 nm. For comparison, OPDs doping by ITIC have extended the effective photodetection range to the full visible spectrum of 380–760 nm. Specifically, the detectivity of obtained OPDs with ratio of 1:0.5:0.5 reached 2.12 × 10^12^ Jones and 2.67 × 10^12^ Jones at 560 nm and 710 nm, respectively. On the one hand, the photodetection range of OPDs have been broadened by the addition of ITIC. On the other hand, the detectivity of optimizing OPDs in the full visible spectrum is higher than that of other OPDs, which is caused by high photocurrent and low dark current at the optimizing ratio of active layer.

## Conclusions

In summary, the high-performance OPDs with full visible light photodetection are fabricated by introducing a non-fullerene acceptor of ITIC into the P3HT:PC_71_BM control system. The three materials form the complementary spectrum, which together effectively realize a broadband photodetector covering whole visible spectrum. Moreover, the OPDs with appropriate ratio of P3HT:PC_71_BM:ITIC exhibit a better photon-harvesting ability, lower dark current, more efficient energy transfer, and more favorable film morphology to improve detectivity. Remarkably, our approach is concise, highly reproducible, and scalable. Our work indicates that choosing suitable non-fullerene electron acceptor and binary system to construct the active layer of complementary light absorption spectrum is an effective method to achieve high-performance broadband OPDs, which will be widespread applicable in the future research.

## Data Availability

All data are fully available without restriction.

## Abbreviations

AFM: Atomic force microscope
BHJ: Bulk heterojunction
Bphen: Bathophenanthroline
D*: Detectivity
EQE: External quantum efficiency
HOMO: The highest occupied molecular orbital
ITIC: 3,9-Bis(2-methylene-(3-(1,1dicyanomethylene)-indanone))-5,5,11,11-tetrakis(4-hexylphenyl)-dithieno[2,3d:2,3′-d′]-s-indaceno[1,2-b:5,6-b′]dithiophene
ITO: Indium tin oxide
*J*_*d*_: Dark current density
*J-V*: The current density-voltage
LUMO: The lowest unoccupied molecular orbital
OPDs: Organic photodetectors
OSCs: Organic solar cells
P3HT: Poly(3-hexylthiophene-2,5-diyl)
PC_71_BM: [6,6]-Phenyl C71-butyric acid methyl ester
PEDOT:PSS: Poly(3,4-ethylenedioxythiophene):polystyrene sulfonate
PL: Steady-state photoluminescence
RMS: Root mean square
UV-Vis: Ultraviolet-visible spectroscopy
